# Peptidylarginine Deiminase 2 Autoantibodies Are Linked to Less Severe Disease in Multiple Sclerosis and Post-treatment Lyme Disease

**DOI:** 10.3389/fneur.2022.874211

**Published:** 2022-06-06

**Authors:** Yaewon Kim, Alison W. Rebman, Tory P. Johnson, Hong Wang, Ting Yang, Carlo Colantuoni, Pavan Bhargava, Michael Levy, Peter A. Calabresi, John N. Aucott, Mark J. Soloski, Erika Darrah

**Affiliations:** ^1^Division of Rheumatology, The Johns Hopkins University School of Medicine, Baltimore, MD, United States; ^2^Lyme Disease Research Center, Division of Rheumatology, The Johns Hopkins University School of Medicine, Baltimore, MD, United States; ^3^Department of Neurology, The Johns Hopkins University School of Medicine, Baltimore, MD, United States; ^4^Department of Neuroscience, Johns Hopkins School of Medicine, Baltimore, MD, United States; ^5^Institute of Genome Sciences, University of Maryland School of Medicine, Baltimore, MD, United States

**Keywords:** PAD2, citrullination, autoantibodies, central nervous system, multiple sclerosis, Lyme disease

## Abstract

**Background:**

Peptidylarginine deiminase 2 (PAD2) mediates the post-translational conversion of arginine residues in proteins to citrullines and is highly expressed in the central nervous system (CNS). Dysregulated PAD2 activity has been implicated in the pathogenesis of several neurologic diseases, including multiple sclerosis (MS). In this study, we sought to define the cellular and regional expression of the gene encoding for PAD2 (i.e. *PADI2*) in the human CNS using publicly available datasets and evaluate whether anti-PAD2 antibodies were present in patients with various neurologic diseases.

**Methods:**

A total of 491 study participants were included in this study: 91 people with MS, 32 people with neuromyelitis optica (NMO), 281 people with post-treatment Lyme disease (PTLD), and 87 healthy controls. To measure *PADI2* expression in the CNS from healthy individuals, publicly available tissue and single cell RNA sequencing data was analyzed. Anti-PAD2 antibodies were measured in the serum of study participants using anti-PAD2 ELISA. Clinical and demographic variables were compared according to anti-PAD2 antibody positivity for the MS and PTLD groups and correlations between anti-PAD2 levels and disease severity were examined.

**Results:**

*PADI2* expression was highest in oligodendrocytes (mean ± SD; 6.4 ± 2.2), followed closely by astrocytes (5.5 ± 2.6), microglia/macrophages (4.5 ± 3.5), and oligodendrocyte precursor cells (3.2 ± 3.3). There was an increased proportion of anti-PAD2 positivity in the MS (19.8%; *p* = 0.007) and PTLD groups (13.9%; *p* = 0.057) relative to the healthy controls (5.7%), and these antibodies were not detected in NMO patients. There was a modest inverse correlation between anti-PAD2 levels and disease severity in people with MS (τ = −0.145, *p* = 0.02), with levels being the highest in those with relapsing-remitting disease. Similarly, there was a modest inverse correlation between anti-PAD2 levels and neurocognitive score (τ = −0.10, *p* = 0.027) in people with PTLD, with difficulty focusing, memory changes, fatigue, and difficulty finding words contributing most strongly to the effect.

**Conclusion:**

*PADI2* expression was observed in diverse regions and cells of the CNS, and anti-PAD2 autoantibodies were associated with less severe symptoms in subsets of patients with MS and PTLD. These data suggest that anti-PAD2 antibodies may attenuate inflammation in diseases of different etiologies, which are united by high *PADI2* expression in the target tissue.

## Introduction

Growing evidence suggests a role for the peptidylarginine deiminase 2 (PAD2) enzyme in the pathogenesis of neurodegenerative, neuroinflammatory, and autoimmune diseases (1, 2). PAD2 belongs to a family of five calcium-dependent enzymes that convert arginine residues in proteins to the non-classical amino acid citrulline, in a process known as citrullination (3). PAD2 is normally expressed in a variety of tissues in the body, with the brain being among the highest PAD2-expressing tissues (4). PAD2 plays important physiological roles in several cellular processes (5), and is known to be expressed by oligodendrocytes where it regulates gene transcription and citrullination of myelin basic protein (MBP) (6, 7).

PAD2 dysregulation and increased expression have been observed in the central nervous system (CNS) of people with various neuroinflammatory and neurodegenerative diseases, including multiple sclerosis (MS) (1, 8). MS is a demyelinating disease of the CNS with both neurodegenerative and autoimmune components (9), and is classified into three general subtypes based on the pattern of disease flare and progression: relapsing-remitting MS (RRMS), secondary progressive MS (SPMS), and primary progressive MS (PPMS). Increased PAD2 and citrullinated protein expression are observed in normal appearing white matter in the CNS of people with MS, thought to be driven by hypomethylation of the PAD2 promoter leading to increased PAD2 expression in these regions (10, 11). Increased citrullination of MBP by PAD2 leads to conformational changes in the myelin sheath, increased protease accessibility, and destabilization, which can disrupt nerve impulses and is hypothesized to reveal new MBP epitopes for immune recognition (8, 12).

PAD2 has also been implicated in playing a pathogenic role in the systemic autoimmune disease rheumatoid arthritis (RA). Many parallels exist between the role of PAD2 in MS and RA, including elevated expression of PAD2 in the target tissue and the striking efficacy of PAD inhibition in reducing disease severity in mouse models of both diseases (10, 13–15). PAD2 is found at high levels in the joint tissue and extracellularly in the synovial fluid from patients with RA (2, 16). Citrullination of a group of proteins by PAD2 is implicated in their targeting by anti-citrullinated antibodies, hallmark serological findings in RA (17). Citrullinated proteins have also been shown to stimulate pro-inflammatory cytokine secretion by macrophages, *via* ligation of toll like receptor 4 (TLR4) (18). Interestingly, we recently found autoantibodies to PAD2 in a subset of people with RA with less severe and progressive joint disease (19), suggesting that anti-PAD2 antibodies may attenuate the pathogenic role of PAD2 in RA.

In this study, we sought to define the cellular and regional expression of the gene encoding for PAD2 (i.e. *PADI2*) in the CNS and evaluate whether anti-PAD2 antibodies were present in patients with MS and other neurologic diseases with known or suspected autoimmune etiology. Neuromyelitis optica (NMO) is a demyelinating disease of the CNS characterized by inflammation of the optic nerve and spinal cord (20). Autoantibodies that target aquaporin-4 are used as biomarkers to facilitate diagnosis in NMO (20), but not all patients are positive, suggesting that other antigens may also be targeted. Post-treatment Lyme disease (PTLD) is a heterogeneous condition of unknown etiology that occurs in a subset of people who are treated for Lyme disease but do not return to baseline health and can have persistent systemic, musculoskeletal, and neurocognitive symptoms (21). The discovery of autoantibodies with reactivity to CNS tissue and observed microglial activation in PTLD suggests that an autoimmune response to CNS antigens may occur in some individuals (22, 23). Although PAD2 has not been previously studied in NMO or PTLD, both are diseases with neurologic and autoimmune components (20, 22), in which PAD2 dysregulation may affect disease pathologies. Understanding the CNS expression and immunologic targeting of PAD2 in neurological diseases has important mechanistic and clinical implications, as defining mechanisms that downregulate PAD2 expression or activity may hold promise for the treatment of these disorders.

## Materials and Methods

### Study Population

Sera from a total of 491 study participants were included in this study. Of these, 91 were people with MS, 32 were people with NMO, 281 were people with PTLD, and 87 were healthy controls. Data and sera from people with MS came from the Johns Hopkins Multiple Sclerosis Center, with recruitment and eligibility criteria described extensively elsewhere (24). Data and sera from people with NMO came from the Johns Hopkins NMO Clinic. Individuals were included in this study if they had a diagnosis of NMO as defined by the 2006 Wingerchuk criteria (25), and all but one individual was positive for aquaporin-4 antibodies. Data and sera from people with PTLD and a subset of the healthy controls (*n* = 22) came from the Studies of Lyme Disease Immunology and Clinical Events (SLICE) at the Johns Hopkins Lyme Disease Research Center, as previously described, with the exception that people with PTLD included in the current study were not required to have 6 months' illness duration (26). Data and sera from the remaining healthy controls (*n* = 65) came from an ongoing observational study of healthy donors at the Johns Hopkins Division of Rheumatology. Healthy volunteers who are not pregnant and who do not have a history of cancer, autoimmune disease, or active tuberculosis/HIV/hepatitis infections were eligible for the study. None of the healthy controls had a known history of Lyme disease. All participants signed written informed consent approved by the Johns Hopkins Institutional Review Board.

Demographic variables measured from all study participants include age, sex, and race. Clinical variables measured from people with MS include duration of illness and disease severity measured by the MS severity score (MSSS), as previously described (27, 28). People with MS were grouped according to their MS type, RRMS (*n* = 41), SPMS (*n* = 31), or PPMS (*n* = 16). Three people with MS did not have a documented MS classification, so were exclude from subtype analysis. Anti-aquaporin 4 antibody status was available for individuals with NMO, but no additional clinical information was available. Clinical variables measured from people with PTLD at the time of the study visit included duration of prior antibiotic treatment, duration of illness from onset of initial Lyme disease, two-tier Lyme disease serologic status (29), neurologic Lyme disease status, defined by medical records confirming Bell's Palsy, neuropathy, meningitis, or encephalitis with positive two-tier serology, and symptom severity. Symptom severity associated with PTLD, including the severity of neurologic symptoms, was measured using PLQS as previously described (26). The researchers were blinded to these clinical variables when conducting the experiments to minimize bias.

### *In silico* Analyses of *PADI2* Expression

Single cell sequencing data was retrieved from Gene Expression Omnibus (GEO) database accession no. GSE67835 (30), which contains sequence data from 466 cells (oligodendrocytes (*n* = 38), astrocytes (*n* = 62), microglia/macrophages (*n* = 16), oligodendrocytes precursor cells (*n* = 18), hybrid cells (*n* = 46), microglia/macrophage (*n* = 16), neurons (*n* = 131), and endothelial cells (*n* = 20). Cells were isolated from human cortical tissue from eight adults and individual cells were classified into the categories above as described (31). Processing and visualization of the data was carried out in the R statistical language as previously described (32, 33). We retrieved selected brain region expression data for *PADI2* from two public data sets. The first was data from five donors with 26 brain regions included in the 2010 Allen Institute for Brain Science, Allen Human Brain Atlas (34). Microarray data from three *PADI2* specific probes were normalized across all brains as previously described (https://help.brain-map.org/display/humanbrain/Documentation). The original search can be reproduced at http://human.brain-map.org/microarray/search/show?exact_match=false&search_term=PADI2&search_type=gene&page_num=0. The second dataset was from The Genotype-Tissue Expression (GTEx) Project. The Genotype-Tissue Expression (GTEx) Project was supported by the Common Fund of the Office of the Director of the National Institutes of Health, and by National Cancer Institute (NCI), National Human Genome Research Institute (NHDRI), National Heart, Lung, and Blood Institute (NHLBI), National Institute on Drug Abuse (NIDA), National Institute of Mental Health (NIMH), and National Institute of Neurological Disorders and Stroke (NINDS). The data used for the analyses described in this manuscript were obtained from the GTEx Portal (https://gtexportal.org/home/) on 09/10/2020 using Ensembl Gene ID ENSG00000117115.12. The 17,382 samples included in this dataset are from 54 tissue regions from 948 donors. Details for the exact number of samples for each brain region can be found here: https://gtexportal.org/home/tissueSummaryPage. Data were normalized as previously described (https://gtexportal.org/home/documentationPage#staticTextAnalysisMethods).

### Anti-PAD2 ELISA

Anti-PAD2 antibodies were measured in the serum of study participants using an in-house generated anti-PAD2 enzyme-linked immunosorbent assay (ELISA), as previously described (19). Briefly, recombinant human PAD2 protein containing N-terminal 6 × histidine and T7 tags was bacterially expressed and purified. The histidine tag was removed by thrombin digestion and 100 ng PAD2 protein was coated into each well of a high-binding polystyrene 96-well EIA plate (Costar) overnight. A dilution of 1:250 patient or healthy control sera was used for the primary antibody and a 1:7,500 dilution of goat anti-human IgG HRP was used for the secondary antibody. An 8-point standard curve was present on each plate comprised of dilutions of a known anti-PAD2 positive serum and was used to calculate anti-PAD2 Arbitrary Units (AU) for each sample. Blank wells coated in PBS alone were used to determine the background binding of each serum and these values were subtracted from those obtained from PAD2-coated wells. The cutoff for positivity was set at 4.5 AU as previously defined (6).

### Statistical Analyses

GraphPad Prism version 8.3.0 and SAS statistical software (version 9.4, SAS Institute, Cary, NC) were used for all statistical analyses and graphs. A *p*-value ≤ 0.05 was considered statistically significant throughout. Brown–Forsythe ANOVA with a Dunnett's T3 correction for multiple hypotheses analyses were performed to compare *PADI2* expression in oligodendrocytes to other cell types. Fisher's exact tests were used to determine if the proportions of anti-PAD2 antibody positive people differed between groups, and median anti-PAD2 antibody levels between each group were compared using analysis of variance (ANOVA, Kruskal–Wallis), corrected for multiple comparisons. Clinical and demographic variables were compared according to anti-PAD2 antibody positivity for the MS and PTLD groups using Student's *t*-tests or Mann–Whitney tests for normally and non-normally distributed variables, Chi-squared tests or Fisher's exact tests for categorical variables, as appropriate. The median anti-PAD2 antibody level in each MS subtype was compared using a one-way ANOVA (Kruskal–Wallis) corrected for multiple comparisons. Correlations between anti-PAD2 antibody levels and MS disease severity or PTLD symptom severity were examined using the Kendall's Tau correlation coefficient.

## Results

### PAD2 Is Expressed Broadly Throughout the CNS

We quantified the expression levels of *PADI2* within specific cells and regions of the CNS by *in silico* analysis of publicly available transcriptomic data (30, 31, 34). This analysis revealed that *PADI2* transcripts are detectable in multiple cell types in the CNS (**Figure 4A**). *PADI2* expression was highest in oligodendrocytes [mean reads per kilobase of transcript per million (RPKM) ± standard deviation; 6.4 ± 2.2], as expected. We compared the expression of *PADI2* in all other cell types to oligodendrocytes by Brown–Forsythe ANOVA with a Dunnett's T3 correction for multiple hypotheses. Astrocytes expressed *PADI2* at similarly high levels as oligodendrocytes (5.5 ± 2.6 RPKM, *p* = 0.38), followed by microglia/macrophages (4.5 ± 3.5 RPKM, *p* = 0.29), which demonstrated bimodal expression of *PADI2*, with one subpopulation of cells expressing high levels of *PADI2* and other low levels. Oligodendrocytes precursor cells (3.2 ± 3.3 RPKM, *p* = 0.006), neurons (0.9 ± 1.8 RPKM, *p* < 0.0001) and endothelial cells (1.3 ± 2.4 RPKM, *p* < 0.0001) were the lowest expressors of *PADI2*. Analysis of the GTEx database indicated that *PADI2* was expressed in multiple regions of the CNS with the highest expression observed in the spinal cord and substantia nigra (**Figure 4B**). Analysis of gene expression data from the Allen Human Brain Atlas (34) showed similar results, with *PADI2* transcripts detectable in multiple regions of the human brain, including the white matter, basal ganglia, midbrain, thalamus, and pons (**Figure 4C**). Together, the data reveal widespread expression of *PADI2* within multiple CNS regions and cell types.

### Anti-PAD2 Antibodies Are Found in People With MS and PTLD

Given the widespread expression of *PADI2* throughout the brain and spinal cord, and the finding that PAD2 is a known autoantigen in RA (19), we reasoned that PAD2 may become a target of the immune responses in individuals with CNS symptoms in whom an autoreactive process has been implicated. To address this hypothesis, we performed an ELISA to determine the prevalence of anti-PAD2 antibodies in the sera of individuals with diseases known or suspected to have an autoimmune process affecting the CNS: MS (*n* = 91), NMO (*n* = 32), and PTLD (*n* = 281). Sera from a group of healthy donors was included as a control population (*n* = 87). Demographic variables for each group including age, sex, and race are shown in Table 1. The average age of the healthy controls was lower than in the disease groups, but age was similar among the MS, NMO, and PTLD groups. In addition, the PTLD group contained more individuals who identified as male and white. Analysis of anti-PAD2 antibodies revealed an increased proportion of anti-PAD2 positivity in the MS (19.8%, 95% CI: 12.9%−29.1%; *p* = 0.007) and PTLD groups (13.9%, 95% CI: 10.3%−18.4%; *p* = 0.057) relative to the healthy controls (5.7%, 95% CI: 2.5%−12.8%; Table 1), with significantly higher median antibody levels in people with MS compared to healthy controls [median (interquartile range) of 1.96 (1.17–4.14) vs. 1.34 (0.78–1.98) anti-PAD2 AU; *p*_*adj*_ = < 0.001; Figure 2]. Patients with NMO had significantly lower levels of anti-PAD2 antibodies [0.21 (0.15–0.46) anti-PAD2 AU] than patients with MS, PTLD, or healthy controls (*p*_*adj*_ < 0.0001 for all; Figure 2). Together, these data revealed that anti-PAD2 antibodies are present in a subset of people with MS and PTLD.

**Table 1:** Demographics and anti-PAD2 positivity.

	**HC**	**MS**	**NMO**	**PTLD**
	(*n* = 87)*	(*n* = 91)*	**(*n* = 32)**	(*n* = 281)*
**Age (years), mean** **±SD**	39.9 ± 13.5	48.8 ± 12.5	49.5 ± 11.3	48.10 ± 15.74
**Male**, ***n*** **(%)**	33 (38.4%)	25 (28.4%)	8 (25.0%)	158 (56.2%)
**White**, ***n*** **(%)**	60 (69.8%)	65 (73.9%)	19 (59.4%)	257* (92.1%)
**Anti-PAD2+**, ***n*** **(%)**	5 (5.7%)	18 (19.8%)	0 (0%)	39 (13.9%)

*MS, multiple sclerosis; NMO, neuromyelitis optica; HC, healthy controls; SD, standard deviation; Anti-PAD2+, anti-PAD2 antibody positive individuals.*

^*^Demographic data was available for 88 people with MS and 86 healthy controls, and race data was available for 279 people with PTLD.

**Figure 1:**
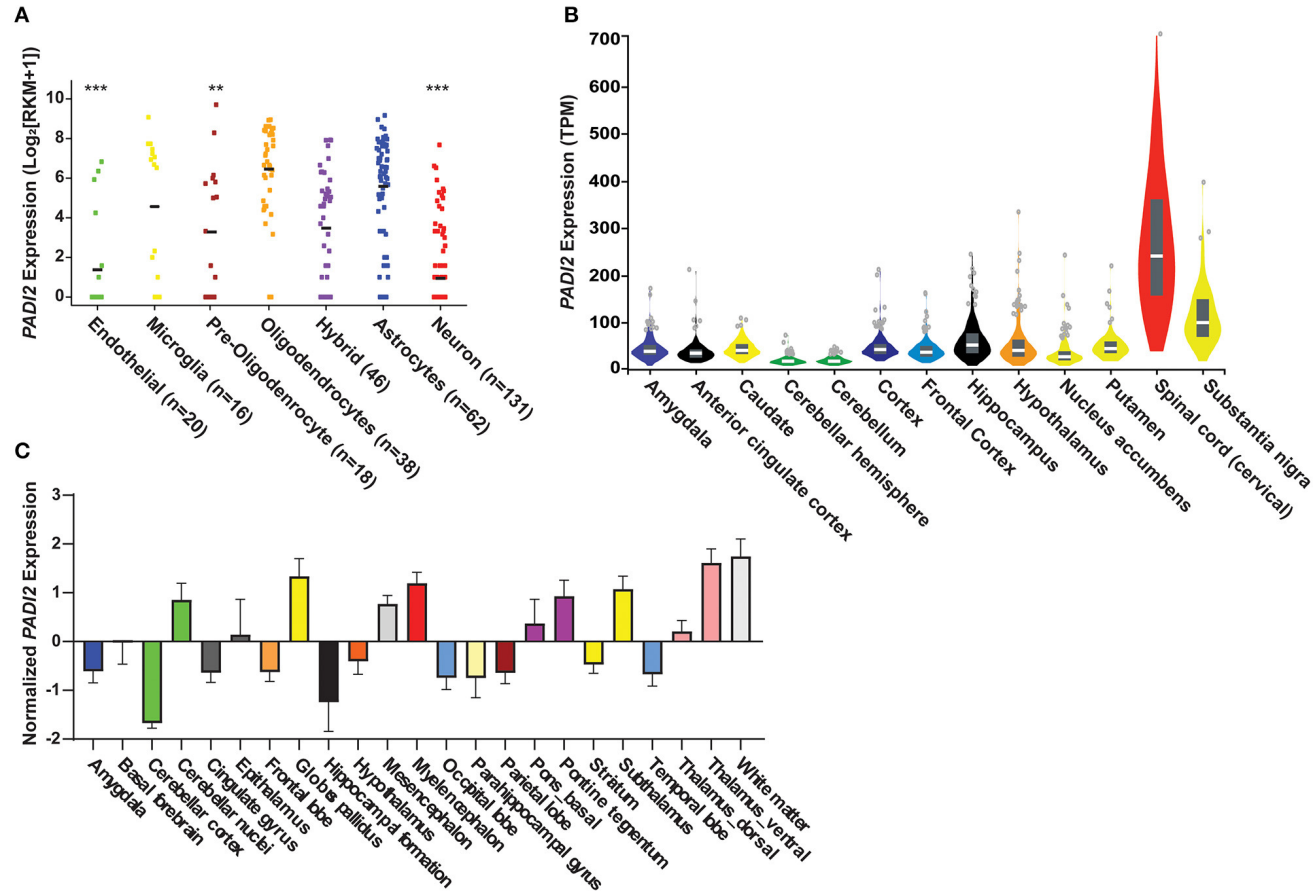
PAD2 transcripts are detectable in glia in the human central nervous system. **(A)** *in silico* analyses of single cell RNA-sequencing data from the GEO human cortex show *PADI2* expression in reads per kilobase of transcript per million (RPKM; *y*-axis) in various cell types (*x*-axis) of the CNS. The number of cells analyzed in each group is provided after the region in paratheses. The mean RPKM of oligodendrocytes was compared to that to the mean of each other cell type in the dataset by ANOVA with Kruskal–Wallis test to corrected for multiple comparisons. ****p-value < 0.0001; **p-value < 0.01. **(B)** *PADI2* expression from bulk RNA sequencing of different CNS regions was downloaded from the GTEx database and gene expression in transcript per million (TPM) is plotted (*y*-axis) for different brain regions and the spinal cord (*x*-axis). Image is modified from (https://gtexportal.org/home/gene/PADI2). **(C)** Transcript expression of *PADI2* in different regions of the brain measured by microarray was downloaded from The Allen Institute. Normalized *PADI2* gene expression (*y*-axis) is shown for multiple brain regions (*x*-axis).

**Figure 2:**
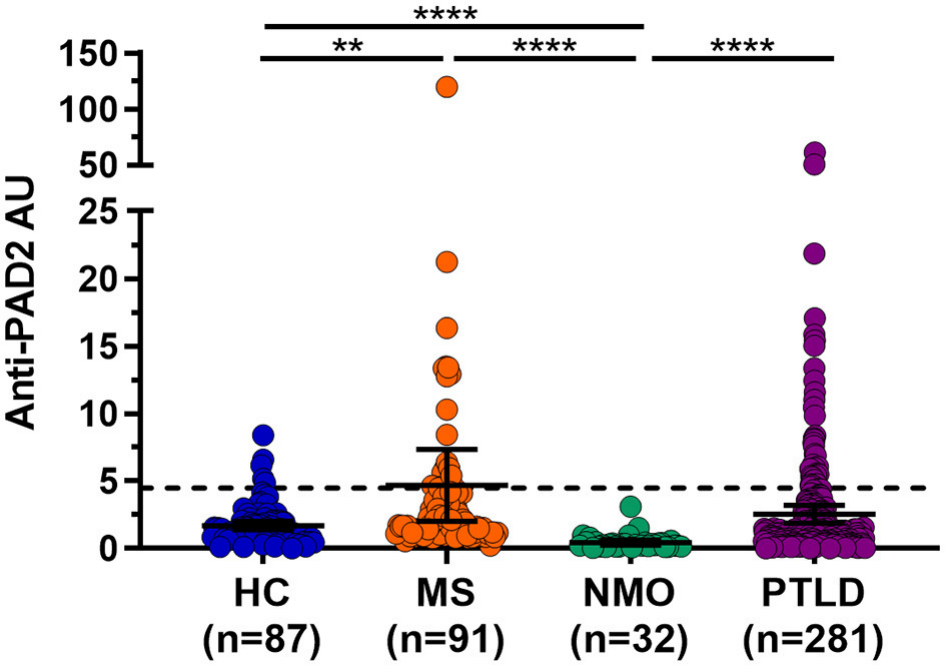
Anti-PAD2 antibody levels in all patient groups. Anti-PAD2 Arbitrary Units (AU) for healthy controls (HC; *n* = 87) or people with multiple sclerosis (MS; *n* = 91), neuromyelitis optica (NMO; *n* = 32), and post-treatment Lyme disease syndrome (PTLD; *n* = 281) as measured by ELISA are shown. The dotted line represents the cutoff value for positivity at 4.5 AU. The median and 95% confidence interval of each group are shown. ****Mann–Whitney *p*-value < 0.0001 and ** < 0.01.

### Anti-PAD2 Antibodies Are Associated With Less Severe MS Symptoms and Relapsing-Remitting Disease

**Figure 3:**
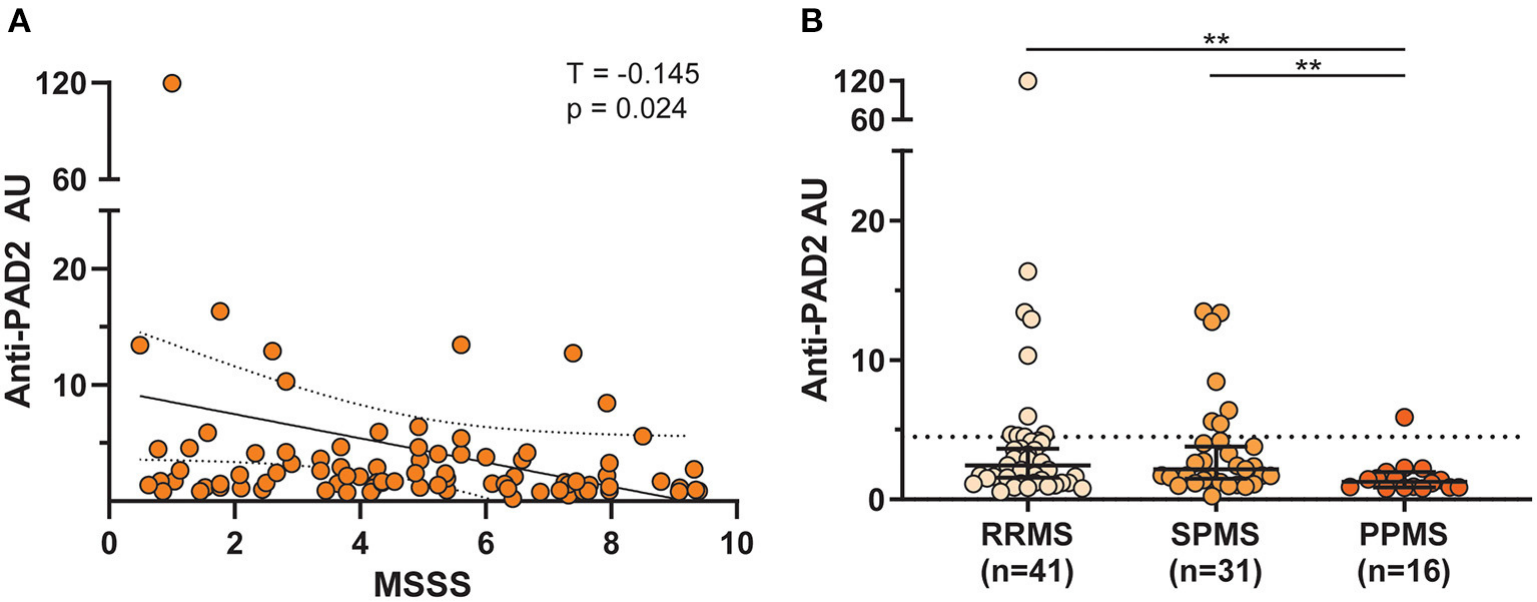
Anti-PAD2 antibody levels by MS subtype and disease severity. **(A)** Scatterplot showing the multiple sclerosis severity score (MSSS) plotted against anti-PAD2 arbitrary units (AU) of all comers with MS with available clinical data (*n* = 88). A univariate analysis was performed between MSSS and anti-PAD2 AU and the Kendall's Tau correlation coefficient (T), *p*-value, trendline (solid black line), and 95% confidence intervals (dotted black lines) are shown. **(B)** Anti-PAD2 Arbitrary Units (AU) in people with RRMS (*n* = 41), SPMS (*n* = 31), and PPMS (*n* = 16) were plotted and compared using a Kruskal–Wallis test adjusted for multiple comparisons. The median and 95% confidence interval are shown. **p < 0.01.

The finding of anti-PAD2 antibodies in a subset of people with MS coupled to the previously reported association of anti-PAD2 antibodies with less severe disease in RA, led us to explore whether anti-PAD2 antibodies were associated with disease severity in MS (Table 2). There were no significant differences in demographic variables, disease duration, or current treatment between anti-PAD2 positive and negative people with MS. However, there was a modest and inverse correlation between anti-PAD2 antibody levels and disease severity, as measured by MSSS in a univariate analysis (τ = −0.145, *p* = 0.02) of all patients with MS, but this trend was not maintained in a multivariable model adjusting for age, sex, and treatment (Figure 3A). In addition, when assessed by MS subtype, anti-PAD2 antibody levels were significantly higher in people with RRMS and SPMS, compared to those with PPMS (Figure 3B). While 22.0% of people with RRMS (9/41) and 22.6% of people with SPMS (7/31) were anti-PAD2 positive, these antibodies were only detected in one individual with PPMS (1/16; 6.3%).

**Table 2:** Characteristics of people with MS according to anti-PAD2 antibody status.

	**Anti-PAD2+**	**Anti-PAD2–**	***p*-Value**
	**(*n* = 17)**	**(*n* = 71)**	
**Age (years), mean** **±SD**	50.8 ± 14.6	48.3 ± 12.0	0.47
**Male**, ***n*** **(%)**	4 (23.5%)	21 (29.6%)	0.78
**White**, ***n*** **(%)**	14 (82.4%)	51 (71.8%)	0.54
Duration of illness*(years), mean **±SD**	16.6 ± 14.9	11.7 ± 9.1	0.10
Treatment*			
**Avonex**, ***n*** **(%)**	2 (11.8%)	3 (4.2%)	0.25
**Betaseron**, ***n*** **(%)**	0 (0%)	1 (1.4%)	1.0
**Rebif**, ***n*** **(%)**	3 (17.6%)	7 (9.9%)	0.40
**Copaxone**, ***n*** **(%)**	6 (35.3%)	20 (28.2%)	0.57
**Gilenya**, ***n*** **(%)**	0 (0%)	1 (1.4%)	1.0
**Lemtrada**, ***n*** **(%)**	0 (0%)	1 (1.4%)	1.0
**Tecfidera**, ***n*** **(%)**	3 (17.6%)	6 (8.5%)	0.37
**Rituximab**, ***n*** **(%)**	0 (0%)	3 (4.2%)	1.0
**Tysabri**, ***n*** **(%)**	2 (11.8%)	12 (16.9%)	1.0

*Anti-PAD2+, anti-PAD2 antibody positive; Anti-PAD2–, anti-PAD2 antibody negative.*

*Duration of illness data was available for n = 87 and treatment data was available for n = 70 people.

### Anti-PAD2 Antibodies Are Associated With Less Severe PTLD Symptoms

**Figure 4:**
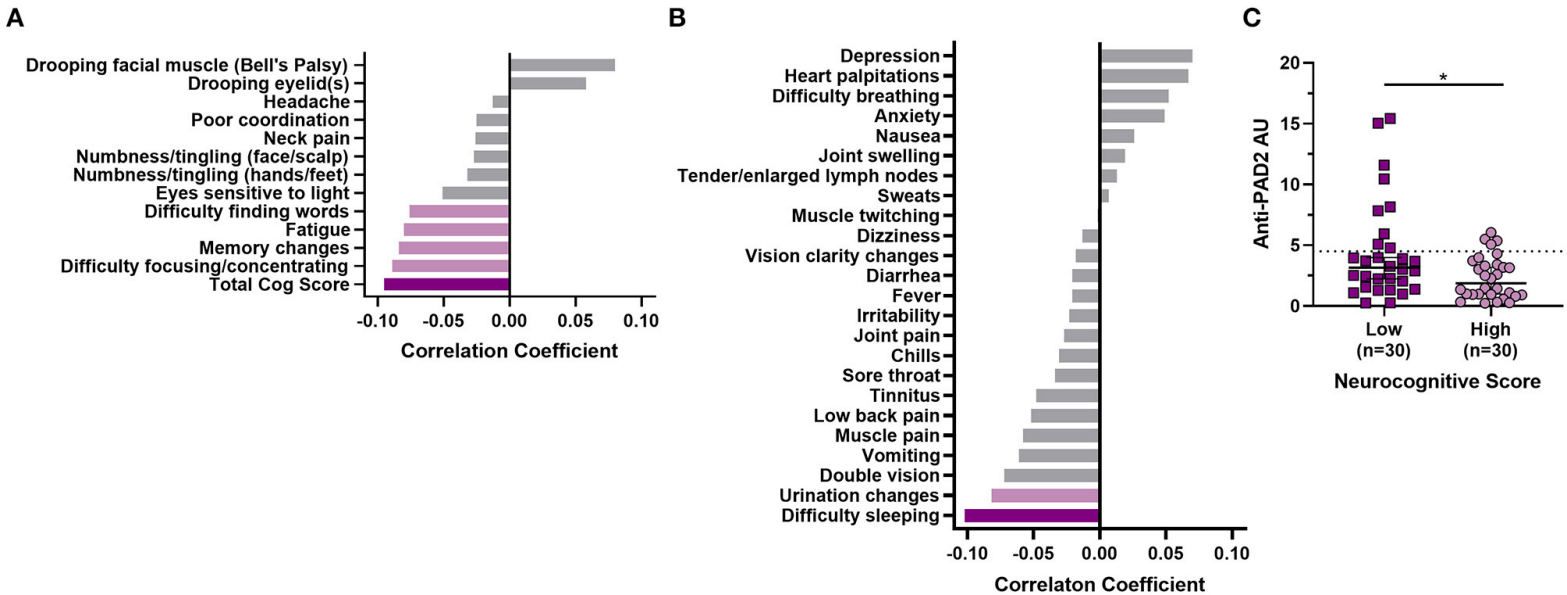
Anti-PAD2 antibody levels in PTLD inversely correlate with symptom severity. **(A)** A bar graph with the Kendall Tau's correlation coefficients for symptoms measured by the PLQS that are included in the neurocognitive score are shown. **(B)** Kendall Tau's correlation coefficients for symptoms measured by the PLQS that are not included in the neurocognitive score are shown. For **(A)** and **(B)**, the dark purple bars indicate symptoms with a significant *p* ≤ 0.05, the light purple bars indicate those with *p* ≤ 0.1, and the gray bars indicate symptoms with *p* > 0.1. **(C)** Anti-PAD2 antibody levels were compared in demographically matched people with PTLD who had the lowest (score <5; *n* = 17; dark purple squares) or highest (score ≥17; *n* = 17, light purple circles) burden of neurocognitive symptoms, as measured by the PLQS. Median anti-PAD2 antibody arbitrary units (AU) were compared by group using the Mann–Whitney *U*-test. *p < 0.05.

Given the clinical heterogeneity in PTLD and the finding that anti-PAD2 antibodies associated with less severe disease in MS, we sought to define whether these autoantibodies associated with neurologic symptom severity in PTLD. When grouped by anti-PAD2 antibody status, anti-PAD2 positive people with PTLD were significantly older, but did not differ by race, sex, two-tier serologic positivity for Lyme disease, duration of illness, duration of antibiotic treatment since the onset of their Lyme disease, or diagnosis of neurologic Lyme disease during the acute infection (Table 3). In a univariate analysis, there was a modest and inverse correlation between anti-PAD2 antibody levels and neurocognitive score (*p* = 0.027), with difficulty focusing or concentrating, memory changes, fatigue, and difficulty finding words contributing most strongly to the effect, but this trend was not maintained in a multivariable model adjusting for age and duration of antibiotic treatment (Figure 4A). Analysis of other systemic symptoms assessed by the PLQS revealed that difficulty sleeping and changes in the frequency or urgency of urination also negatively correlated with anti-PAD2 antibodies in PTLD (Figure 4B). Given that age positively correlated with anti-PAD2 antibodies, we performed a case-control study with 30 individuals with PTLD who had the lowest scores on the PLQS (<5) and 30 individuals with the highest neurological scores (≥17) matched for age, sex, and disease duration. Consistent with the previous observations, anti-PAD2 antibody levels were significantly higher in patients with a lower burden of neurocognitive symptoms (*p* = 0.036).

**Table 3:** Characteristics of people with PTLD according to anti-PAD2 antibody status.

	**Anti-PAD2+** **(*n* = 39)**	**Anti-PAD2–** **(*n* = 242)**	***p*-Value**
**Age (years), mean** **±SD**	52.82 ± 15.44 (18.00, 79.00)	47.34 ± 15.68 (18.00, 82.00)	0.043
**Male** ***n*** **(%)**	20 (51.3%)	138 (57.0%)	0.619
White*, ***n*** **(%)**	34 (87.2%)	223 (92.9%)	0.208
Positive two-tier Lyme disease serology*, ***n*** **(%)**	18 (46.2%)	101(42.3%)	0.779
**Duration of illness (years), mean** **±SD**	3.53 ± 5.23 (0.13, 27.68)	3.13 ± 3.98 (0.06, 28.59)	0.579
**Duration of antibiotic treatment (years), mean** **±SD**	0.36 ± 0.53 (0.04, 2.46)	0.26 ± 0.41 (0.01, 3.12)	0.174
**Neurologic Lyme**, ***n*** **(%)**	1 (2.6%)	20 (8.3%)	0.328

Anti-PAD2+, anti-PAD2 antibody positive; Anti-PAD–, anti-PAD2 antibody negative; SD, standard deviation. ^*^Race data was available for n = 240 anti-PAD2- people and positive two-tier Lyme disease serology data was available for n = 239 anti-PAD2- people.

## Discussion

Our study sought to define the expression of *PADI2* in the CNS at the cellular and regional level using publicly available datasets and to determine whether people with CNS pathologies generate autoantibodies to the PAD2 protein. We observed widespread expression of *PADI2* in several cell types and regions within the CNS and a higher prevalence of anti-PAD2 antibodies in people with MS and PTLD compared to either healthy controls or people with NMO. We found an enrichment of anti-PAD2 antibodies in people with relapsing subtypes of MS (RRMS and SPMS), and a modest inverse correlation of anti-PAD2 antibody levels with disease severity. Surprisingly, we also found anti-PAD2 antibodies in a subset of PTLD patients and again observed a modest association with less severe disease. It is interesting to note the lack of anti-PAD2 antibodies in people with NMO, suggesting that the pathologic process is distinct and does not result in autoimmunity to PAD2. Indeed, although NMO is a demyelinating disease of the CNS that shares several clinical features with MS, autoantibodies to aquaporin-4 are diagnostic of the disease and have been identified as a key pathogenic mediator of CNS damage (20), mechanistically setting it apart from MS. Our findings in MS and PTLD parallel our previous observation that anti-PAD2 antibodies are associated with milder symptoms in people with RA, and suggest that anti-PAD2 antibodies may play a role in attenuating inflammation across a spectrum of disorders.

The presence of anti-PAD2 antibodies in subsets of people with RRMS and SPMS and association with a less severe disease in MS suggests that these antibodies may define a mechanistically distinct group of patients in which PAD2 plays a pathogenic role. An important criterion in the diagnosis of MS is the presence of oligoclonal bands (OCBs), indicative of immunoglobins of the IgG subclass, in the cerebrospinal fluid (CSF) (35). OCBs are present in 95% of people with MS and are regarded as important indicators for the diagnosis of MS. However, the antigens targeted by these antibodies, which carry the potential to provide significant insight into MS etiology, remain largely unknown (36). Considering the known role of PAD2 in MS pathogenesis and our discovery that PAD2 is a target antigen in a subset of people with MS, it will be important to define whether PAD2 is targeted by autoantibodies present in the CSF of patients. In addition, longitudinal studies in larger MS cohorts are needed to interrogate whether anti-PAD2 antibodies associate with less severe or progressive disease at the individual level.

The finding that anti-PAD2 antibodies are present in a subset of people with PTLD suggests an underlying immunological component in PTLD pathology in these individuals. Although PTLD is an idiosyncratic disease of unknown etiology, one long-standing hypothesis is that the bacterium that causes Lyme disease, *Borrelia burgdorferi*, may trigger an autoimmune response resulting in persistent symptoms even after successful antibiotic treatment (37). Interestingly, a recent positron emission tomography (PET) imaging study, using a radiotracer specific for activated microglia and reactive astrocytes, demonstrated high levels of signal across multiple brain regions in people with PTLD compared to healthy controls (22). This finding suggested diffuse immune activation in the brains of people with PTLD that may contribute to the development of neurocognitive symptoms (26). Our finding of high PAD2 expression in both microglia and astrocytes as well as in multiple regions of the CNS parallels this observation and suggests that further study of the role of PAD2 in PTLD is warranted. Longitudinal studies are needed to examine whether anti-PAD2 antibodies are present early in Lyme disease infection, are able to predict the development of PTLD in people with acute Lyme, and are associated with changes in the clinical progression of PTLD.

The widespread expression of *PADI2* in the CNS may provide an explanation for why anti-PAD2 antibodies correlate with less severe symptoms in both MS and PTLD. Our working model is that PAD2 dysregulation in cells expressing high levels of PAD2 may result in higher PAD2 activity that contributes to immune activation in the CNS *via* two primary mechanisms: (1) activation of microglia and astrocytes, and (2) destabilization of the myelin sheath. Macrophages, microglia, and astrocytes have all be shown to express TLR4, and citrullinated proteins have been shown to activate pro-inflammatory cytokine production by macrophages *via* ligation of TLR4 (18). In addition, hyperactivation of PAD2 in oligodendrocytes may promote demyelination *via* citrullination of MBP leading to destabilization of the myelin sheath, increased degradation by proteases, and revelation of neoepitopes for targeting by autoreactive cells (12, 38). Together, these changes may drive CNS inflammation, including the development of autoreactivity to CNS antigens and development of neurocognitive symptoms. Our data suggest that a subset of people with CNS disease develop anti-PAD2 antibodies, which may attenuate PAD2-dependent inflammation and lead to a reduction of symptoms. It will be important to address critical aspects of this model in future studies.

## Conclusion

We have found circulating anti-PAD2 antibodies in subsets of people with MS and PTLD, which associate with milder neurologic symptoms. Combined with our published data in RA, our current findings reveal that anti-PAD2 antibodies, present in a subset of individuals, associate with less severe symptoms in diseases united by high *PADI2* expression in the target tissue (i.e. the synovium in RA and the CNS in MS and PTLD). While it remains unknown whether PAD2 expression correlates with PAD2 activity at these sites, the implications of our results are that anti-PAD2 antibodies may hold potential as a novel prognostic biomarker to define less severe subsets and inform future development of mechanism-guided therapies that target PAD2 in these diseases.

## Data Availability Statement

The original contributions presented in the study are included in the article/supplementary materials, further inquiries can be directed to the corresponding author/s.

## Ethics Statement

Studies were approved by the Johns Hopkins Institutional Review Board and all participants signed written informed consent approved by the Johns Hopkins Institutional Review Board (IRB00066509, IRB00098663, IRB00035457, NA_00071455, and NA_00011170).

## Author Contributions

YK performed PAD2 ELISAs, analyzed resulting data, and prepared associated figures and drafted the manuscript. AR and TY analyzed and interpreted PTLD clinical data. TJ analyzed and interpreted cellular and tissue transcriptomics data and prepared corresponding figures. HW prepared PAD2 antigen and optimized PAD2 ELISAs. CC curated and analyzed transcriptomics data. PB analyzed and interpreted MS clinical data. ML provided NMO sera and clinical data. PC provided MS sera and clinical data. JA provided PTLD sera and clinical data. MJS contributed to curation of the SLICE biobank and hypothesis generation. ED led overall study design, data interpretation, and manuscript preparation. All authors contributed to writing the manuscript and approved the final version for publication.

## Funding

This work was supported by funding from the office of the Assistant Secretary of Defense for Health Affairs through the Peer Reviewed Medical Research Program under Award No. W81XWH2010284, Global Lyme Alliance, Steven and Alexandra Cohen Foundation, NIH NIAMS P30AR053503, and NINDS R37NS041435. The content is solely the responsibility of the authors and does not necessarily represent the official views of the NIH. PB is supported by a Career Transition Award from the National MS Society.

## Conflict of Interest

ED has a licensed agreement and provisional patent for the use of anti-PAD2 antibodies to identify patient subsets in rheumatoid arthritis and other diseases and has received research funds from Pfizer, Bristol Myers Squib, Celgene outside of this current work. ML received consulting fees from Alexion, Viela BioGenentech/Roche/Chugai, Quest Diagnostics, Mitsubishi Pharma and UCB Pharmaceuticals outside of this current work. The remaining authors declare that the research was conducted in the absence of any commercial or financial relationships that could be construed as a potential conflict of interest.

## Publisher's Note

All claims expressed in this article are solely those of the authors and do not necessarily represent those of their affiliated organizations, or those of the publisher, the editors and the reviewers. Any product that may be evaluated in this article, or claim that may be made by its manufacturer, is not guaranteed or endorsed by the publisher.

## Abbreviations

PTLD: post-treatment Lyme disease
PLQS: Post-Lyme Questionnaire of Symptoms
SLICE: Studies of Lyme disease Immunology and Clinical Events
